# Porokeratotic Eccrine Ostial and Dermal Duct Nevus

**DOI:** 10.1155/2013/953840

**Published:** 2013-11-04

**Authors:** Mona Masoumeh Naraghi, Azita Nikoo, Azadeh Goodarzi

**Affiliations:** ^1^Department of Pathology, Razi Hospital, Tehran University of Medical Sciences, P.O. Box 14515-736, Tehran, Iran; ^2^Department of Dermatology, Razi Hospital, Tehran University of Medical Sciences, P.O. Box 14515-736, Tehran, Iran

## Abstract

PEODDN is a rare benign cutaneous disorder that clinically resembles comedo nevus but favors the palms and soles, where pilosebaceous follicles are absent. Widespread involvement along Blaschko's lines can also occur. It is a disorder of keratinization involving the intraepidermal eccrine duct (acrosyringium) and is characterized by eccrine hamartoma and cornoid lamellation in pathology. The patient is a 29-year-old man with an 8-year history of pruritic skin lesions on his right lateral ankle. In the pathologic examination, multiple small epidermal invagination with overlying parakeratotic cornoid lamellation, loss of granular layer, and few dyskeratotic cells at the base of epidermal invagination are revealed. After clinic-pathologic correlation, the diagnosis of porokeratotic eccrine ostial and dermal duct nevus (PEODDN) was made. Late-onset and rare clinical presentation as pruritic lesion are the characteristic features that make this patient an extraordinary presentation of PEODDN.

## 1. Introduction 

The term Porokeratotic Eccrine Ostial and Dermal Duct Nevus (PEODDN) was first described in 1980 by Abell and Read [1]. However, it was first described by Marsden et al. in 1979 [2]. PEODDN is a very rare skin condition which is classified as porokeratotic dermatoses. It is characterized by cornoid lamella which is a column of parakeratotic cells and is associated with dyskeratosis in the spinous layer as well as reduction in the number of granular zone cells. It is in close association with subjacent acrosyringia [3]. Here, we report a case with this rare condition.

## 2. Case Report 

**Figure 1:**
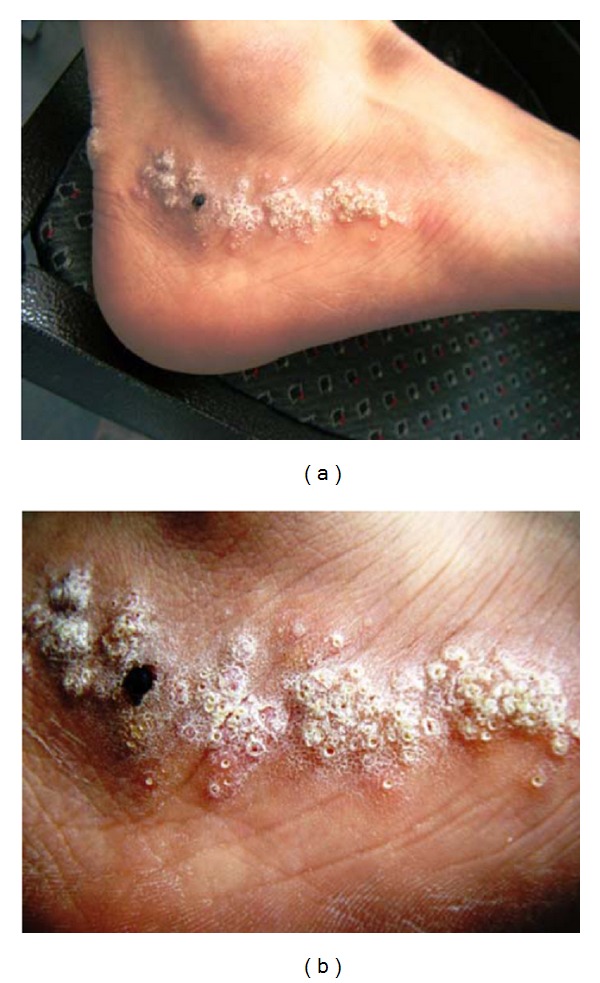
Linearly distributed multiple keratotic papules of a similar size on the lower part of the right lateral ankle. He had no personal history of extracutaneous disease and no family history of similar lesions.

A 29-year-old Iranian gentleman was referred to the Dermatology Department of RAZI Skin Hospital with severe pruritic skin lesions on his right lateral ankle that had presented since 8 years ago. Physical examination revealed multiple keratotic papules of similar size in a linear distribution forming verrucous plaques over the lower part of his right lateral ankle (Figure 1).

**Figure 2:**
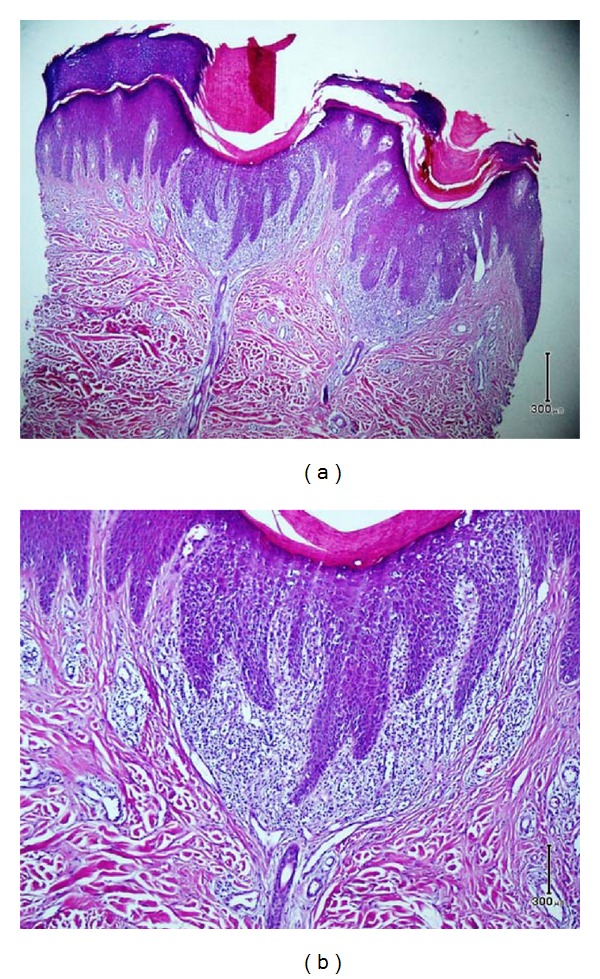
Small epidermal invagination with overlying parakeratotic cornoid lamellation and underlying, slightly tortuous, eccrine duct nearby the epidermis. Loss of granular layer and few dyskeratotic cells were evident at the base of epidermal invagination (Figure 2(a), H&E ×100), (Figure 2(b), H&E ×400).

One of the papules was removed by punch biopsy and examined under the microscope. Pathologic evaluation revealed multiple small epidermal invagination with overlying parakeratotic cornoid lamellation and underlying, slightly tortuous, eccrine duct nearby the epidermis. Loss of granular layer and few dyskeratotic cells were evident at the base of epidermal invagination (Figure 2).

## 3.  Discussion 

POEDDN is firstly reported by Marsden et al. as a comedo nevus of the palm in 1979 [2]. According to our best knowledge, there are about 41 case reports and 6 literature reviews about that all around the world. Many of them present at birth or at young ages although some may occur in adults or even in the elderly [3]. It is usually asymptomatic, although it may be accompanied by a mild pruritus, hyperhidrosis, or anhidrosis. Association with other conditions is rare and includes neurological problems, scoliosis, palmoplantar keratoderma, onychodysplasia, alopecia, and hyperthyroidism [4–6].

Etiologically, it has been proposed that the invagination of the epidermis may result from an abnormal clone of epidermal cells which leads to the formation of cornoid lamella [7]. Another hypothesis suggests that the invagination is a dilated acrosyringeal and dermal duct which is keratin-plugged [8]. It is also supposed that Porokeratotic Eccrine Nevus may be caused by somatic connexin 26 mutations [9].

Histopathology is the mainstay of diagnosis; cornoid lamella with the involvement of acrosyringia is pathognomonic for PEODDN. It is usually associated with the dilation of eccrine duct. Differential diagnoses include porokeratosis plantaris discreta, inflammatory linear verrucous epidermal nevus, nevus comedonicus, linear epidermal nevus, linear psoriasis, spiny keratoderma, linear porokeratosis, congenital unilateral punctate porokeratosis, and porokeratosis of Mibelli [10, 11].

In conclusion, although PEODDN is often an early-onset and asymptomatic or mildly pruritic lesion, we describe a case of late-onset PEODDN with severe pruritus on his right lateral ankle. Because of the rarity of this condition, each diagnosed case of PEODDN should be reported to enhance our knowledge regarding this condition. We would like to emphasis that late-onset pruritic lesions with blaschkoid distribution could be one of the clinical presentations of PEODDN. 

## References

[1] Abell E, Read SI (1980). Porokeratotic eccrine ostial and dermal duct naevus. *British Journal of Dermatology*.

[2] Marsden RA, Fleming K, Dawber RPR (1979). Comedo naevus of the palm—a sweat duct naevus?. *British Journal of Dermatology*.

[3] Wang NS, Meola T, Orlow SJ, Kamino H (2009). Porokeratotic eccrine ostial and dermal duct nevus: a report of 2 cases and review of the literature. *American Journal of Dermatopathology*.

[4] Jamora MJJ, Celis MA (2008). Generalized porokeratotic eccrine ostial and dermal duct nevus associated with deafness. *Journal of the American Academy of Dermatology*.

[5] Rasi A, Tajziechi L (2008). Late-onset porokeratotic eccrine ostial and dermal duct nevus associated with sensory polyneuropathy and hyperthyroidism. *Archives of Iranian Medicine*.

[6] Kroumpouzos G, Stefanato CM, Wilkel CS, Bogaars H, Bhawan J (1999). Systematized porokeratotic eccrine and hair follicle naevus: report of a case and review of the literature. *British Journal of Dermatology*.

[7] Bergman R, Lichtig C, Cohen A, Friedman-Birnbaum R (1992). Porokeratotic eccrine ostial and dermal duct nevus: an abnormally keratinizing epidermal invagination or a dilated, porokeratotically plugged acrosyringium and dermal duct?. *American Journal of Dermatopathology*.

[8] Stoof TJ, Starink TM, Nieboer C (1989). Porokeratotic eccrine ostial and dermal duct nevus. Report of a case of adult onset. *Journal of the American Academy of Dermatology*.

[9] Easton JA, Donnelly S, Kamps MAF (2012). Porokeratotic eccrine nevus may be caused by somatic connexin26 mutations. *Journal of Investigative Dermatology*.

[10] Bolognia JL, Jorizzo JL, Schaffer JV (2012). *Dermatology*.

[11] McKee PH (2012). *McKee’s Pathology of the Skin*.

